# Heat Shock Proteins: Pathogenic Role in Atherosclerosis and Potential Therapeutic Implications

**DOI:** 10.1155/2012/502813

**Published:** 2012-12-11

**Authors:** Arman Kilic, Kaushik Mandal

**Affiliations:** Division of Cardiac Surgery, Department of Surgery, Johns Hopkins Hospital, Baltimore, MD 21287, USA

## Abstract

Heat shock proteins (HSPs) are a highly conserved group of proteins that are constitutively expressed and function as molecular chaperones, aiding in protein folding and preventing the accumulation of misfolded proteins. In the arterial wall, HSPs have a protective role under normal physiologic conditions. In disease states, however, HSPs expressed on the vascular endothelial cell surface can act as targets for detrimental autoimmunity due to their highly conserved sequences. Developing therapeutic strategies for atherosclerosis based on HSPs is challenged by the need to balance such physiologic and pathologic roles of these proteins. This paper summarizes the role of HSPs in normal vascular wall processes as well as in the development and progression of atherosclerosis. The potential implications of HSPs in clinical therapies for atherosclerosis are also discussed.

## 1. Introduction

Heat shock proteins (HSPs) were first discovered as being expressed in response to increased temperature, as the name suggests [1]. This family of proteins is highly conserved, displaying high sequence homology between prokaryotes and eukaryotes and between different species [2]. The highly conserved nature of HSPs is a reflection of their essential role in protective mechanisms from stress conditions. At the intracellular level, HSPs act as molecular chaperones and assist in the folding of misfolded proteins, thereby preventing their aggregation. At the extracellular level, HSPs can elicit immunogenic responses. These basic functions of HSPs are evident in the human arterial wall, where HSPs have been shown to be important mediators of protective pathways as well as targets for autoimmunity leading to atherosclerosis [3, 4].

Although the diverse roles of HSPs in normal arterial physiology as well as in atherosclerosis have been discussed in prior reviews, the body of literature at both the basic science and clinical levels has expanded exponentially in this field in recent years [3–5]. As such, the purpose of this paper is to provide an updated overview of our understanding of the role of HSPs in atherosclerosis. In addition, an updated review of the potential clinical implications of HSPs in atherosclerosis-directed therapy is provided as well.

## 2. Methods

We performed our literature search using MEDLINE, with no limits regarding date of publication. The search terms used were “heat shock proteins” and “atherosclerosis”. Limits included articles in English only.

## 3. Results 

### 3.1. Role of HSPs in Normal Physiologic Processes of the Arterial Wall

The arterial wall is undoubtedly a dynamic structure that continually responds to stresses in its environment [6]. HSPs, which are classified according to their molecular weight, have been implicated in a variety of physiologic processes in the normal arterial wall that are aimed at protecting these structures from such stresses (Table 1). The principal function of HSPs is in protein folding and unfolding. Also, by modulating misfolded proteins, HSPs prevent their aggregation within the cell. Specific subtypes of HSPs, however, exhibit different secondary functions or mechanisms of function.

HSP60, for instance, has been shown to have roles in polypeptide assembly and protein translocation across membranes, in addition to protein folding [7]. Its diverse roles are reflected in the fact that it is found in several intracellular compartments, including the nucleus, cytoplasm, endoplasmic reticulum, and mitochondria [8]. HSP10 acts as a cofactor for HSP60 [9]. HSP27 is a protein associated with estrogen receptor-*β*, a receptor that is expressed in vascular smooth muscle and endothelium [10]. This HSP subtype has been shown to be protective against atherosclerosis by competing with the uptake of lipids [11]. HSP70 is a two-domain structure consisting of a 45 kilodalton and 25 kilodalton unit, which has several significant roles at the intracellular level, including protein folding, translocation across membranes, and degradation of proteins. HSP70 has also been shown to have anti-inflammatory effects by reducing the activation of the proinflammatory gene transcription factor, nuclear factor kappa-light-chain-enhancer of activated B cells (NF-*κβ*) [12]. Finally, HSP90 is a molecular chaperone involved in the folding and activation of several proteins integral to transcriptional regulation and signal transduction [13].

### 3.2. The Link between Autoimmunity to HSPs and the Development of Atherosclerosis

#### 3.2.1. HSP60

HSP60 is the most relevant and well-studied HSP subtype with regards to autoimmunity and development of atherosclerosis. Indeed, earlier studies formulated the hypothesis that T-cell-mediated and humoral immune responses to HSP60 in endothelial regions subject to hemodynamic stress were the initiating event in atherosclerosis [14]. Autoimmunity to HSP60 exists in normal healthy individuals. This stems from its high level of sequence homology with bacterial HSPs, reflecting its conservation through evolution.

#### 3.2.2. HSP65

The HSP60 family also includes HSP65, which is the mycobacterial homologue of mammalian HSP60 [15]. Indeed, more than 95% sequence homology exists between HSP60 from various bacteria including mycobacterial HSP65, and 50%–55% homology exists between human HSP60 and mycobacterial HSP65 with upwards of 70% homology in highly conserved regions [16].

#### 3.2.3. Stress-Related Expression of HSP60 on Vascular Endothelial Cell Surfaces

**Figure 1:**
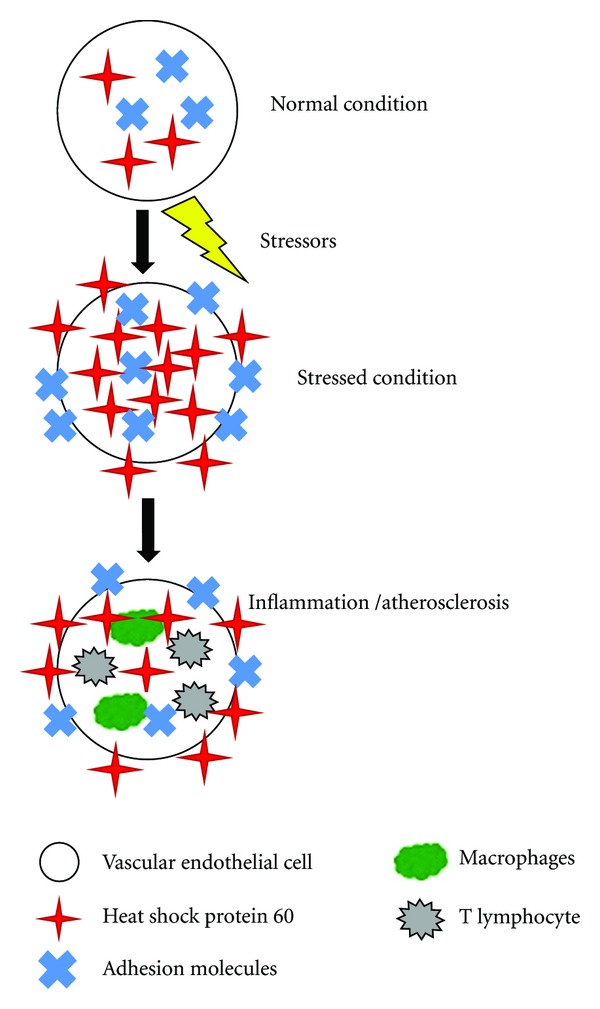
Concept of autoimmunity towards heat shock protein 60 and the development of atherosclerosis. Under normal conditions, heat shock protein 60 is located intracellularly and is not expressed on the vascular endothelial cell surface. Under stressed conditions, heat shock protein 60 and various adhesion molecules are upregulated and expressed on the cell surface. This leads to inflammation and the development of atherosclerosis.

Under normal conditions, HSP60 is not expressed on the vascular endothelial cell surface. However, under stressed conditions including the traditional risk factors for atherosclerosis, mitochondrial HSP60 is translocated to the cytoplasm and then to the cell surface (Figure 1) [17]. Preexisting immunity to HSP60 then leads to its targeting, with resulting inflammatory cascades and progression of atherosclerosis. In addition to the risk factors for atherosclerosis, the other stressors that can induce expression of HSP60 on the endothelial cell surface include infections, mechanical stress, and temperature change. It is important to mention that in addition to inducing the expression of HSP60 on the vascular endothelial cell surface, the various stressors also induce the expression of adhesion molecules on the cell surface, including VCAM-1, ELAM-1, and ICAM-1 [18]. HSP60 itself induces E-selectin, VCAM-1, ICAM-1, and IL-6 production within the endothelial cell [19]. The upregulated expression of these molecules is an important contributor to the HSP60-directed autoimmune pathogenesis of atherosclerosis.

#### 3.2.4. Immune Cell Types in Atherosclerotic Lesions

With regards to immune cell subtypes, CD4+ T cells are present in the highest concentration in the earliest phases of atherosclerosis. Furthermore, the strongest T-cell reactions against HSP60 are found in intralesional T cells, which display an oligoclonally restricted receptor phenotype, as compared to extralesional peripheral T cells which have weaker reactions to HSP60 and display polyclonal phenotypes [20]. A study of young clinically healthy males found that T-cell reactivity against HSP60 was an independent risk factor for early intima-media thickening (Table 2) [21]. 

The role of B cells in the development of atherosclerosis is less clearly understood. Several studies have demonstrated progression of atherosclerotic disease with B-cell depletion, whereas other studies demonstrated reduction in the disease with B-cell depletion [22–24]. These differing results may be due to the presence of both atherogenesis-promoting as well as atherogenesis-inhibiting antibodies/mediators. Similar to the different roles of these antibodies, other inflammatory mediators have also been shown to either promote or inhibit atherosclerosis. IL-1*β*, IL-8, IL-12, IL-18, MCP-1, leukotriene P4, and IFN-*γ* have been demonstrated as proatherogenic mediators whereas IL-4, IL-10, PDGF-*β*, and TGF-*β* have been shown to be antiatherogenic [25].

#### 3.2.5. Soluble HSP60

In addition to being expressed on the endothelial cell surface, HSP60 can be shed into the circulation in a soluble form under stressed conditions. A study of 826 human patients found that levels of soluble HSP60 were significantly elevated in patients with carotid atherosclerosis [26]. The authors postulated that the release of HSP60 from cells may be mediated by infectious agents. More specifically, chlamydiae are known to exhibit both nonlytic and lytic infective phases, and during the latter, the human host cell releases both its own HSP60 as well as chlamydial HSP60. This postulate is supported by evidence that both HSP60 subtypes exist in high concentrations in atherosclerotic lesions, and that soluble HSP60 levels correlate with anti-Chlamydial antibody titers [27]. More recent studies have also shown the association between elevated levels of soluble HSP subtypes and various cardiovascular diseases (Table 3) [15, 28–32].

#### 3.2.6. Serum Antibodies to HSP60 and HSP65

Similar to soluble HSP60, prior studies have also demonstrated elevated serum antibody levels to HSP65, which is the mycobacterial homolog of human HSP60 [33]. A followup study also showed that anti-HSP65 antibodies remained consistently elevated over several years in humans with progressive atherosclerosis [34]. Furthermore, levels of anti-HSP65 antibodies correlated strongly with antibody titers to *Chlamydia pneumoniae* and *Helicobacter pylori*, suggesting an infectious role [35]. Coronary events were observed more frequently in patients that had high HSP60 IgA levels coupled with high titers of antibodies to *Chlamydia pneumoniae* and high C-reactive protein levels [36]. However, a polymerase chain reaction study of 40 atherosclerotic human patients and 20 nonatherosclerotic human controls found similar detection rates of *Chlamydia pneumoniae*, *Mycoplasma pneumoniae*, *Helicobacter pylori*, herpes simplex virus, and cytomegalovirus in the aortic wall, which does not support the infectious etiology hypothesis for atherosclerosis [37].

#### 3.2.7. Establishing Causality in HSP60/65 Antibody-Mediated Atherosclerosis

Although soluble HSP60 and antibodies to HSP60/65 have been shown to be elevated in human patients with atherosclerosis, it is unclear whether this simply represents an association or whether a causal relationship exists. Administration of a murine monoclonal antibody (II-13) to amino acid residues 288 to 366 of HSP60 induced atherosclerosis in apolipoprotein E-deficient mice [38]. II-13 injection resulted in endothelial cell damage, leukocyte attachment, and accumulation of macrophages and smooth muscle cells in lesions. The same study demonstrated that isolating anti-HSP60 antibodies from humans with coronary atherosclerosis and injecting them into apolipoprotein E-deficient mice caused significant increases in aortic atherosclerotic lesions [38]. Passive transfer of T cells from mice immunized with mycobacterial HSP65 to nonimmunized mice led to the development of atherosclerosis in the nonimmunized cohort [39].

A study of 120 normocholesterolemic rabbits found that those immunized with recombinant mycobacterial HSP65 had increased atherosclerosis [40]. In the rabbits that were fed a cholesterol-rich diet in addition to being immunized with HSP65, the atherosclerotic lesions were even more severe. A followup investigation by the same group demonstrated that the early atherosclerotic lesions induced by HSP65 could be inhibited by T-cell depletion using an anti-CD3 monoclonal antibody [41]. 

#### 3.2.8. HSP10

HSP10 is an important cofactor for HSP60 [9]. The significant interplay between these HSP subtypes is further evidenced by the fact that their genes are localized in a head-to-head manner on chromosome 2, separated by a bidirectional promoter [42]. Similar to HSP60, the overexpression of HSP10 is met with an overexpression of BcL-2 and Bcl-xL [43]. These molecules protect vascular endothelial cells from TNF-mediated apoptosis in addition to inhibiting activation of NF-*κβ* and thereby inhibiting the upregulation of proinflammatory genes [44]. The antiapoptotic roles of HSP10 are evidenced by the fact that transfecting doxorubicin-treated cardiomyocytes with HSP10 and HSP60 by an adenoviral vector suppresses apoptosis and resulting cardiomyopathy [43]. A study of antibodies to HSP10 of *Chlamydia pneumoniae *in patients with coronary artery disease failed to demonstrate significant differences in levels versus controls; however, the importance of HSP10 to the development of atherosclerosis may indeed lie in its genetic and physiologic link to HSP60 [45].

#### 3.2.9. HSP27

Emerging data has implicated HSP27 in the pathogenesis of atherosclerosis. A study of human atherosclerotic plaques revealed an increase in expression of HSP27 in normal-appearing vessel adjacent to the plaque, with decreased levels in the plaque itself [46]. HSP27 phosphorylation was decreased in both plaque and adjacent vessel compared to reference vessel. And finally, when the investigators examined HSP27 levels in plasma, they found that in patients with acute coronary syndrome, levels of HSP27 were increased and found to correlate with levels of HSP70, C-reactive protein, and CD40L [46].

Another study similarly found that HSP27 release was significantly decreased in atherosclerotic plaques [47]. Circulating levels of soluble HSP27 were also significantly decreased in patients with carotid stenosis compared with healthy controls [47]. A study of 22 heart transplant recipients found that those with cardiac allograft vasculopathy had significantly reduced levels of phosphorylated HSP27 in biopsy samples as compared to those recipients without vasculopathy [48]. The decreased expression of HSP27 within plaques may be related to its degradation by enhanced proteolytic pathways which are known to be important contributors to vascular remodeling [49].

HSP27 may also offer protection from atherosclerosis due to its role in plaque stability. A proteomic analysis of stable versus unstable human carotid artery atherosclerotic plaques found reduced levels of HSP27 in unstable lesions [50]. Moreover, at the molecular level, phosphorylated HSP27 is known to be a regulator of actin filament dynamics [51]. Furthermore, HSP27 may modulate the effects of plasmin and other extracellular mediators of apoptosis in vascular smooth muscle cells, a process which has been shown to lead to plaque instability through the weakening of the fibrous cap of the atheroma with potential plaque rupture and resultant atherothrombosis [52]. 

In addition, HSP27 has been demonstrated *in vitro* to be released into the extracellular space in response to various stimuli, including estrogen or acetylated low-density lipoprotein, where it binds the scavenger receptor A to prevent low-density lipoprotein uptake and foam cell formation [53]. HSP27, which is an estrogen receptor-*β* associated protein, also modulates estrogen signaling and may have additional atheroprotective functions via this mechanism [54]. Increased estrogen receptor-*β* expression has indeed been noted in both males as well as pre- and postmenopausal females with atherosclerosis [55, 56].

In an apolipoprotein E-deficient animal model, overexpression of human HSP27 resulted in a 35% reduction in aortic atherosclerosis in female, but not male, mice [53]. Serum levels of HSP27 were over tenfold higher in females as compared to males, again using the model of HSP27-overexpressing apolipoprotein-E deficient mice. Circulating HSP27 levels demonstrated a strong inverse correlation with atherosclerotic lesion area in both female and male mice [53].

#### 3.2.10. HSP70

In early atherosclerosis, dendritic cells exclusively overexpress HSP70 as well as HLA-DR and CD1d, the latter being a unique molecule used in lipid antigen presentation [57]. These HSP70-expressing dendritic cells also frequently interact with T cells within the arterial wall and therefore may be responsible for presenting lipid antigens to them. Unlike the early stages, several immune cell types, including macrophages, smooth muscle cells, monocytes, and dendritic cells, have been shown to overexpress HSP70 in advanced atherosclerosis [57]. Furthermore, a gene expression profiling analysis revealed that two HSP70 family members were expressed within aortic atherosclerotic lesions but not within nonlesional tissue [58]. 

Another study found that oxidized low-density lipoprotein stimulated the expression of HSP70 and that supernatants from oxidized low-density lipoprotein-treated macrophages could induce both IL-1*β* and IL-12 secretion in naïve macrophages [59]. Furthermore, this latter effect on cytokine production was inhibited by inhibiting HSP70 transcription or secretion. Extracellular HSP70 could therefore be an important inducer of cytokine expression and inflammation. 

HSP70 may also have anti-inflammatory roles. In one immunization study, a peptide sequence of myobacterial HSP70 was found to induce the production of IL-10 by peptide-specific T cells, a phenomenon that was also seen with T cells responsive to the whole HSP70 protein [60]. IL-10 is known to be a potent anti-inflammatory cytokine, and indeed, its production was found in the prior study to prevent arthritis. Another study found that HSP70 attenuated NF-*κβ* activation and its associated proinflammatory gene upregulation [61]. The potentially protective roles of HSP70 were further supported by a study of 421 blood samples from human subjects which found that high levels of HSP70 were associated with low risk of coronary artery disease [62]. Another study also found low plasma levels of HSP70 in patients with atherosclerosis, with activated neutrophils being a potential source for proteases involved in HSP70 degradation [63].

HSP70 may also be implicated in the calcification of blood vessels. HSP70 was found to enhance bone morphogenetic protein-4-induced proliferation in endothelial cells and to enhance bone morphogenetic protein-induced calcium deposition in vascular cells [64]. The same study found that HSP70 mediated the IL-6 procalcific effect on vascular cells. Levels of HSP70, bone morphogenetic protein-4, and IL-6 were all elevated within the aortic wall as well as the serum in a mouse model of atherosclerosis [64]. Antibodies to HSP70 diminished this procalcific effect. 

#### 3.2.11. HSP90

A study of human carotid atherosclerosis demonstrated overexpression of HSP90 in both plaque and serum as compared to healthy controls [65]. Moreover, plaque-derived and circulating T cells from patients with atherosclerosis proliferated in response to HSP90 whereas cells from controls did not. Finally, HSP90-specific T cells expressed both proinflammatory and anti-inflammatory cytokines, implying a dichotomous role [65].

Another study of human atherosclerotic plaques found that the expression of HSP90 was associated with plaque instability in advanced lesions [66]. Inhibitors of HSP90 also reduced atherosclerosis-related inflammation in their analysis. Another investigation by the same group found that HSP90 inhibitors interfere with oxidative stress by reducing pro-oxidative factors in experimental atherosclerosis [67]. 

The potential anti-inflammatory therapeutic benefits of HSP90 inhibitors have been demonstrated in other diseases as well. In a mouse model of systemic lupus erythematosus, HSP90 was found to have a potential role in regulating T-cell differentiation and activation, and its inhibition was associated with reduced inflammation [68]. In a murine sepsis model, the administration of HSP90 inhibitors resulted in reduced systemic and pulmonary inflammatory markers compared to controls as well as improved lung function and survival [69].

### 3.3. Clinical Implications

#### 3.3.1. Screening, Diagnosis, and Prognosis

There are several clinical implications related to HSPs and their role in atherosclerosis. One potential clinical application would be to exploit the presence of HSP antibodies for screening at-risk patients to detect significant atherosclerosis. A study of 750 human subjects demonstrated the correlation between HSP65 antibody titers and advanced carotid atherosclerotic lesions [29]. Another study similarly showed that anti-HSP65 antibody titers correlated strongly with severity of coronary atherosclerosis [70]. In both of these studies, these findings persisted after adjusting for potential confounders such as patient age and smoking history. In addition to identifying atherosclerosis, there may be a role in identifying patients who have suffered from myocardial infarction. HSP70, for instance, was found to be rapidly released in significant quantities following an acute myocardial infarction in 24 patients, highlighting its potential as a marker for myocardial damage [28]. 

Screening patients based on titers could be a useful strategy for the detection of significant atherosclerosis, although the sensitivity and specificity of such a test are unknown, as are the cost implications. Such blood tests may have more utility in directing diagnostic workup for coronary artery disease, particularly in patients with marginal indications for further testing. In these intermediate risk patients, antibody titers could be used as further risk stratification, with those patients with high antibody levels undergoing further workup and those with undetectable or low levels continuing to undergo clinical surveillance. 

HSPs may also have a role in prognosis [71]. In a study of 750 patients, HSP65 antibody titers were found to predict 5-year mortality [29]. Another investigation found that among 79 individuals with angiographic evidence of coronary artery disease, anti-HSP65 titers were higher among those with future cardiovascular events than in those without [72]. In addition, in a study of 588 consecutive emergency admissions of patients with acute chest pain of suspected cardiac origin, those with high anti-HSP60 titers had a worse one-year prognosis [73]. 

#### 3.3.2. Treatment

In addition to its potential screening, diagnostic, and prognostic roles, there may be potential utility for HSPs in the treatment of atherosclerosis (Table 4). There has been growing interest in the role of HSP vaccination in this effort. Mice lacking the receptor for low-density lipoprotein were nasally vaccinated with HSP65 in one study and subject to cholesterol-rich diets [74]. Vaccinated mice had significant decreases in atherosclerotic plaque size, a reduced number of T cells, and an increased IL-10 expression, the latter of which is an antiatherogenic mediator. Another study also found that inducing oral tolerance by feeding low-density lipoprotein receptor-deficient mice with HSP65 led to IL-4 (antiatherogenic) production and a reduction in atherosclerosis [75]. Both of these studies suggest that inducing shifts from a Th1 to Th2 phenotype could be associated with protection from atherosclerosis. 

There are clinical concerns with such vaccines given the high sequence homology between human and bacterial HSPs. Inducing tolerance may result in increased susceptibility to serious infections. Despite these concerns, there have been phase I and II trials in the realm of cancer that have demonstrated the safety of similar vaccines. In metastatic melanoma, for instance, vaccination with autologous tumor-derived HSP peptide complexes resulted in only mild toxicity in some patients, limited mostly to local erythema and induration at the injection site [76]. The safety of HSP vaccines directed at atherosclerosis remains to be elucidated, however, as does the longer term safety profiles of these vaccines in general.

There have been several recent studies published regarding potential HSP-related therapies for atherosclerosis. One study examined the effect of a vaccine that targets HSP65 and cholesterol ester transfer protein simultaneously and found more protective IL-10, less adverse IFN-*γ*, less serum, low-density lipoprotein, and a significant reduction in aortic atherosclerotic plaque burden in vaccine-treated rabbits [77]. An expert opinion piece highlighted the potential of combining regulatory T-cell-targeted therapies using dominant HSP peptides with current biological therapies for autoimmune and inflammatory conditions such as atherosclerosis [78]. Another study in a diabetic rat model concluded a potential therapeutic role of glutamine-induced HSP70 expression, a finding that was observed in both serum and aortic wall [79]. 

There have also been several recent studies regarding the potential of HSP-related therapies in diseases other than atherosclerosis (Table 4). One such study demonstrated that KW-2478, which is a novel HSP90 inhibitor, enhanced the antitumor effects of bortezomib (a proteasome inhibitor) both *in vitro* and *in vivo* in multiple myeloma [80]. Another study in acute leukemia also demonstrated significant anti-tumor effects with an HSP70 inhibitor both alone and in combination with other antineoplastic agents [81]. Additionally, an analysis of flavaglines, which are a family of natural products with known neuroprotective properties, found that they also have cardioprotective effects in the setting of doxorubicin therapy, and that this effect is mediated by HSP27 [82]. Finally, remote ischemic preconditioning was found to reduce spinal cord damage in a rat model likely through HSP70 overexpression [83].

## 4. Conclusions

A growing body of evidence in both animal models and human subjects has implicated autoimmunity towards HSPs as a potential pathogenic mechanism for the development of atherosclerosis. Ongoing and future studies that further elucidate the mechanisms whereby HSPs, infection, and immune response pathways interact and lead to the common pathway of atherosclerosis will be essential to developing more specific and potentially safer novel therapies for this devastating disease process. A better understanding of the functions of HSPs in other pathologies such as cancer may also be useful in advancing our knowledge of the role of this important family of molecules in atherosclerosis and their potential therapeutic utility.

## Figures and Tables

**Table 1:** Functions of heat shock proteins.

Heat shock protein molecular weight (kilodaltons)	Function	Pathologic associations
60	Protein folding	Atherosclerosis
	Protein unfolding	Rheumatoid arthritis
	Polypeptide assembly	Systemic sclerosis
	Protein translocation across membranes	Schizophrenia
		Diabetes mellitus

10	Cofactor for HSP 60	Cardiovascular disease

27	Competes for uptake with lipids	Atherosclerosis
	Estrogen receptor-*β*-associated protein	

70	Protein folding	Atherosclerosis
	Protein unfolding	Leprosy
	Degradation of misfolded or denatured proteins	Tuberculosis
	Assembly of new proteins	
	Translocation of proteins across membranes	

90	Molecular chaperone involved in protein folding and activation	Atherosclerosis
		Systemic lupus erythematosus

**Table 2:** Logistic regression analysis for the impact of various risk factors on high vascular intima-media thickness in a study of 141 young (17- or 18-year old) white males (see [21]).

Risk factor	Odds ratio (95% CI)	*P* value
Cigarette smoking	3.58 (1.34–9.54)	0.0108
High-density lipoprotein level	0.56 (0.36–0.89)	0.0144
Alcohol consumption	0.51 (0.30–0.87)	0.0133
Diastolic blood pressure	1.61 (1.03–2.52)	0.0374
Maximum expiratory flow at 50% vital capacity	0.52 (0.33–0.82)	0.0047
HSP60 stimulation index	2.18 (1.32–3.60)	0.0023
HSP60 antibody titer	1.52 (1.00–2.31)	0.0514

Odds ratios were calculated based on a 1 standard deviation unit change in the given variable.

**Table 3:** Soluble heat shock proteins and their association with cardiovascular diseases.

Soluble heat shock protein subtype	Number of patients	Cardiovascular disease	Study finding	Reference
HSP70	24	Acute myocardial infarction	Soluble HSP70 is released into the circulation after an acute myocardial infarction	[28]
HSP60	684	Carotid atherosclerosis	Levels of soluble HSP60 are associated with early carotid atherosclerosis	[29]
HSP70	52 cases 20 controls	Acute myocardial infarction	Levels of soluble HSP70 are associated with progression of heart failure after acute myocardial infarction	[30]
HSP60, HSP72	88 cases 44 controls	Idiopathic left ventricular dysfunction	Levels of soluble HSP60 and HSP72 correlate with severity of cardiac and microvascular dysfunction in patients with idiopathic left ventricular dysfunction	[15]
HSP70	167	Congestive heart failure	Levels of soluble HSP70 are associated with severity of heart failure in patients with congestive heart failure	[31]
HSP60	1003 cases 1003 controls	Coronary artery disease	Levels of soluble HSP60 correlate with the presence of coronary artery disease	[32]

**Table 4:** Summary of studies on potential heat shock protein-related treatments for various diseases.

Study	Disease	Subjects	Study summary and major findings
Maron et al. [74]	Atherosclerosis	Mice	Nasal vaccination with HSP65 resulted in a significant decrease in the size of atherosclerotic plaques, a reduced number of T cells, and an increased IL-10 expression
Harats et al. [75]	Atherosclerosis	Mice	Oral tolerance induced with HSP65 led to a reduction in atherosclerosis
Jun et al. [77]	Atherosclerosis	Rabbits	Vaccine targeting HSP65 and cholesterol ester transfer protein reduced low-density lipoprotein levels and atherosclerotic burden
Ishii et al. [80]	Multiple myeloma	Human	The addition of an HSP90 inhibitor enhanced the antitumor activity of a proteasome inhibitor both *in vitro* and *in vivo *
Kaiser et al. [81]	Acute leukemia	Human	HSP70 inhibitor displayed antileukemic effects both alone and in combination with other antineoplastic agents
